# A Fatty Surprise: Lipogranulomatous Inflammation in a Peripancreatic Node

**DOI:** 10.7759/cureus.95597

**Published:** 2025-10-28

**Authors:** Muhammed Vally, Vikash Lala, Adam Mahomed, Martin Hale, Washington Mudini

**Affiliations:** 1 Department of Gastroenterology, Wits University Donald Gordon Medical Centre, University of the Witwatersrand, Johannesburg, ZAF; 2 Department of Pathology, Wits University Donald Gordon Medical Centre, University of the Witwatersrand, Johannesburg, ZAF

**Keywords:** endoscopic ultrasound, incidental finding, lipogranulomatous inflammation, lymph node, masld

## Abstract

A 62-year-old man with a history of metabolic dysfunction-associated steatotic liver disease underwent an endoscopic ultrasound (EUS) for further evaluation. Imaging revealed a 2.7 × 2.2 cm hyperechoic lymph node located between the pancreatic head and the common bile duct. The node appeared steatotic in nature. EUS-guided biopsy demonstrated benign lymphoid tissue with lympholipogranulomatous inflammation and no evidence of malignancy. While such nodes are often incidental, this type of lipogranulomatous inflammation is an uncommon finding on EUS, with limited literature describing its clinical implications.

## Introduction

Lymph nodes with lipogranulomatous inflammation are rare and often incidental histological findings, especially in the setting of endoscopic ultrasound (EUS) [1]. While benign lymphadenopathy is a relatively common observation during EUS, lympholipogranulomatous changes are infrequently encountered and may mimic malignancy or inflammatory pathology on imaging [1]. This creates a diagnostic challenge, particularly when identified in anatomically complex regions such as the peri-pancreatic area.

These lesions may occur in the context of prior infections, systemic inflammatory states or metabolic disorders [1]. In our case, the node was located between the pancreatic head and the common bile duct (CBD), an area where any abnormal finding raises concern for pancreaticobiliary malignancy. The presence of background metabolic dysfunction-associated steatotic liver disease (MASLD) and a previous history of renal infection/nephronia may contribute to the node's atypical appearance and inflammatory nature. Recognising this entity is important to avoid unnecessary surgical interventions or oncologic therapies [2].

Here, we present a rare case of a lymph node with lipogranulomatous inflammation incidentally discovered during EUS in a patient with known MASLD and a history of nephronia. This case illustrates the importance of correlating EUS findings with histopathology and highlights a unique benign differential for hyperechoic lymphadenopathy in the pancreatic region.

## Case presentation

A 62-year-old man with a background of metabolic syndrome, including hypertension, dyslipidaemia and increased abdominal circumference, presented to the emergency department with a two-day history of fever, chills and dysuria. On examination, he had diffuse abdominal tenderness with localised right-sided renal angle tenderness. He was otherwise clinically stable, and the remainder of the systemic examination was unremarkable.

Initial laboratory investigations revealed a mildly elevated C-reactive protein (CRP), with evidence of mild acute kidney injury (Table 1). Specifically, his CRP was 67 mg/L (reference: <5 mg/L) and urea was 6.9 mmol/L (reference: 2.9-8.2 mmol/L), with a creatinine of 114 µmol/L (reference: 80-115 µmol/L) and an eGFR of 59 mL/minute/1.73 m², consistent with a mild reduction in renal function. Furthermore, liver function tests were within normal limits, although the GGT was slightly high at 61 IU/L (reference: 0-64 IU/L). The lipid profile was also within normal limits overall, and the serum ACE was 69.3 IU/L (reference: 20-70), which was not suggestive of sarcoidosis. In addition, the virology screen was negative for hepatitis A, B and C, as well as HIV. Urine microscopy and culture on the same date showed no significant pyuria or bacteriuria, and no growth was reported, arguing against an active urinary tract infection at that time (Table 2).

**Table 1:** Summary of initial laboratory investigations Initial results showing an elevated C-reactive protein and mild renal dysfunction, consistent with an acute infectious and inflammatory process TCO₂: total carbon dioxide; eGFR: estimated glomerular filtration rate; CKD-EPI: Chronic Kidney Disease Epidemiology Collaboration; ALT: alanine transaminase; AST: aspartate aminotransferase; GGT: gamma-glutamyl transferase; ALP: alkaline phosphatase; ACE: angiotensin-converting enzyme; CRP: C-reactive protein; LDL: low-density lipoprotein; HDL: high-density lipoprotein

Tests	Results	Units	Reference range
Full blood count			
Haemoglobin	17	g/dL	13.8-18.8
Platelet count	404	×10⁹/L	150-450
White cell count	6.36	×10⁹/L	4.0-12.0
Urea and electrolytes			
Sodium	134	mmol/L	136-145
Potassium	3.8	mmol/L	3.5-5.1
Chloride	100	mmol/L	98-107
Bicarbonate (TCO₂)	18	mmol/L	21-29
Urea	6.9	mmol/L	2.9-8.2
Creatinine	114	µmol/L	80-115
eGFR (CKD-EPI)	59	mL/minute/1.73 m²	-
Liver function tests			
Albumin (serum)	38	g/L	35-50
ALT	37	IU/L	<50
AST	40	IU/L	<50
GGT	61	IU/L	0-64
ALP	80	IU/L	53-128
Total bilirubin	24	µmol/L	3-26
Conjugated bilirubin	10	µmol/L	2-7
Total protein	77	g/L	60-80
Serum ACE			
ACE	69.3	IU/L	20.0-70.0
Inflammatory markers			
CRP	67	mg/L	0.0-5.0
Lipogram			
Total cholesterol	4.2	mmol/L	<5.0
LDL cholesterol	2.7	mmol/L	<3.0
HDL cholesterol	1.5	mmol/L	>1.0
Triglycerides	2	mmol/L	<1.7
Virology			
Hepatitis screen			
Hepatitis A IgM	Negative	-	-
Hepatitis B surface antigen	Negative	-	-
Hepatitis B core Ab	Negative	-	-
Hepatitis B surface Ab	Nonimmune (0.00 mIU/mL)	-	-
Hepatitis C Ab	Negative	-	-
HIV	Negative	-	-

**Table 2:** Urine microscopy and culture findings Urine microscopy and culture showed no significant pyuria or bacteriuria, and no growth was reported

Urine microscopy and culture	Results	Units	Normal limit
Leucocytes	<5	/µL	<25
Red blood cells	<5	/µL	<10
Dysmorphic red cells	0	%	0
Epithelial cells	Not observed	-	-
Bacteria	Not observed	-	-
Casts	Not observed	-	-
Crystals	Not observed	-	-
Yeast cells	Not observed	-	-
Culture	No growth	-	-

**Figure 1:**
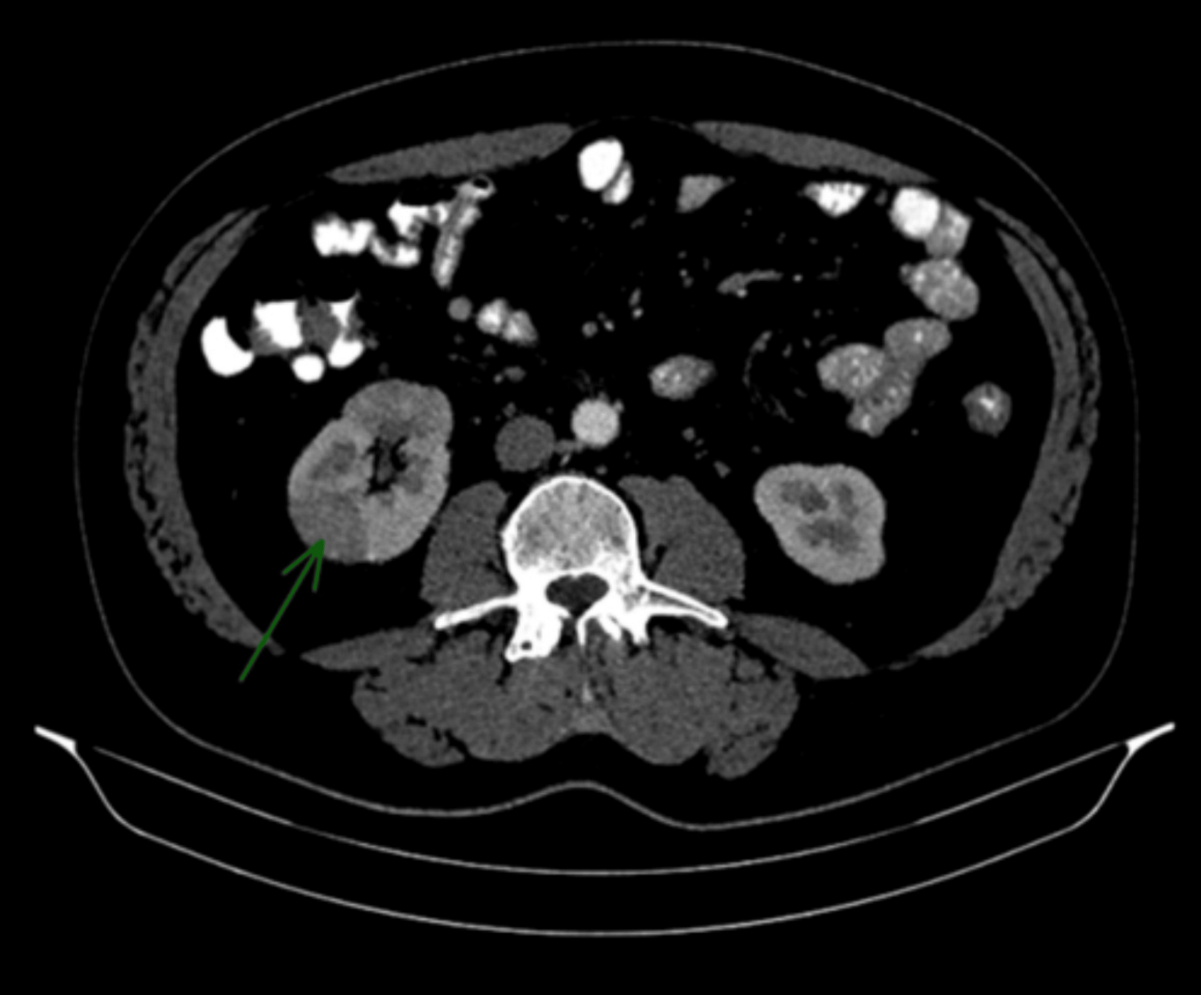
Contrast-enhanced CT of the abdomen (axial view) Right-sided focal nephronia, characterized by ill-defined, low-attenuation cortical changes without abscess formation

**Figure 2:**
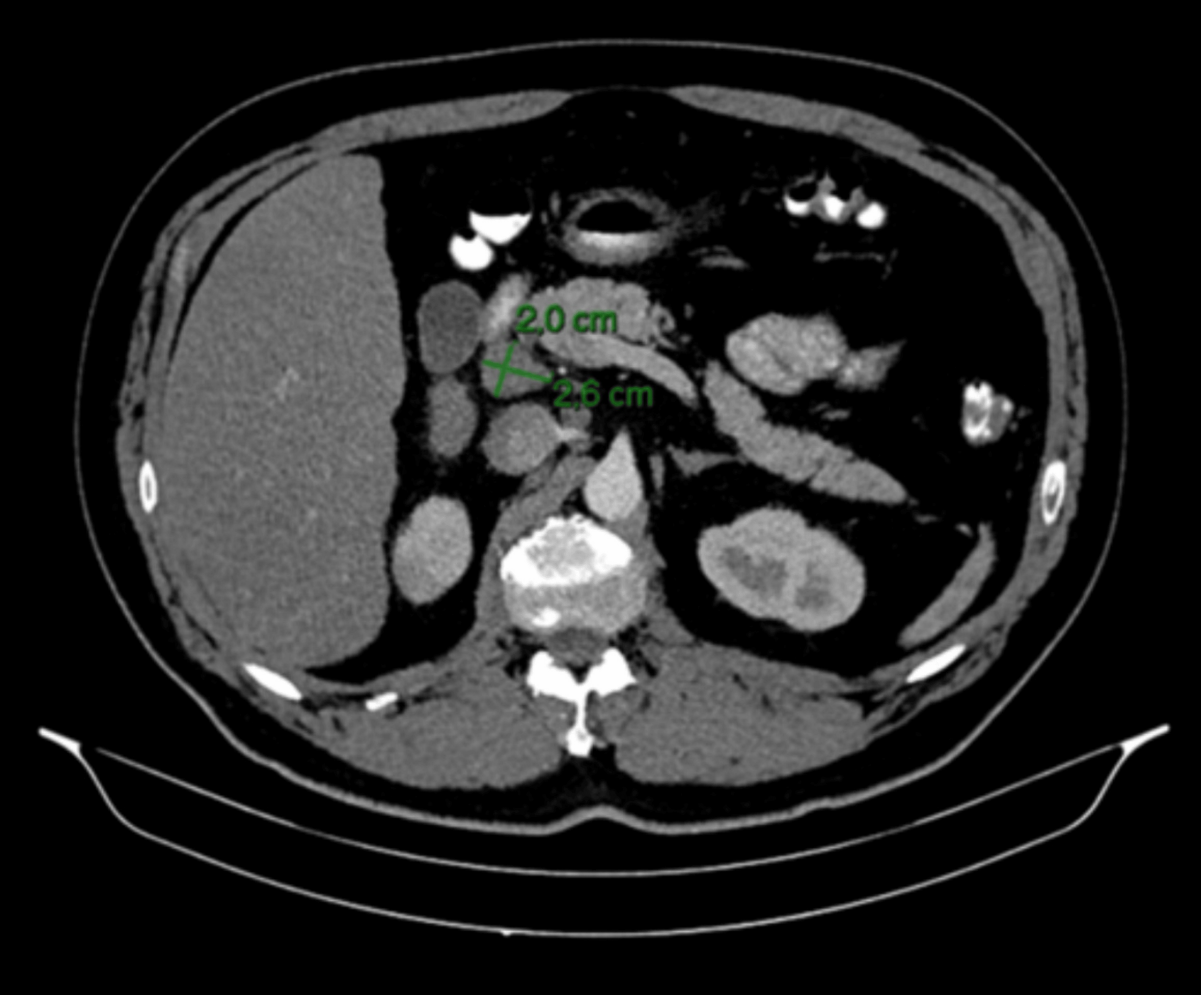
CT abdomen (axial view), showing enlarged lymph node adjacent to the pancreatic head (2.6 × 2.0 cm) in the right upper quadrant

**Figure 3:**
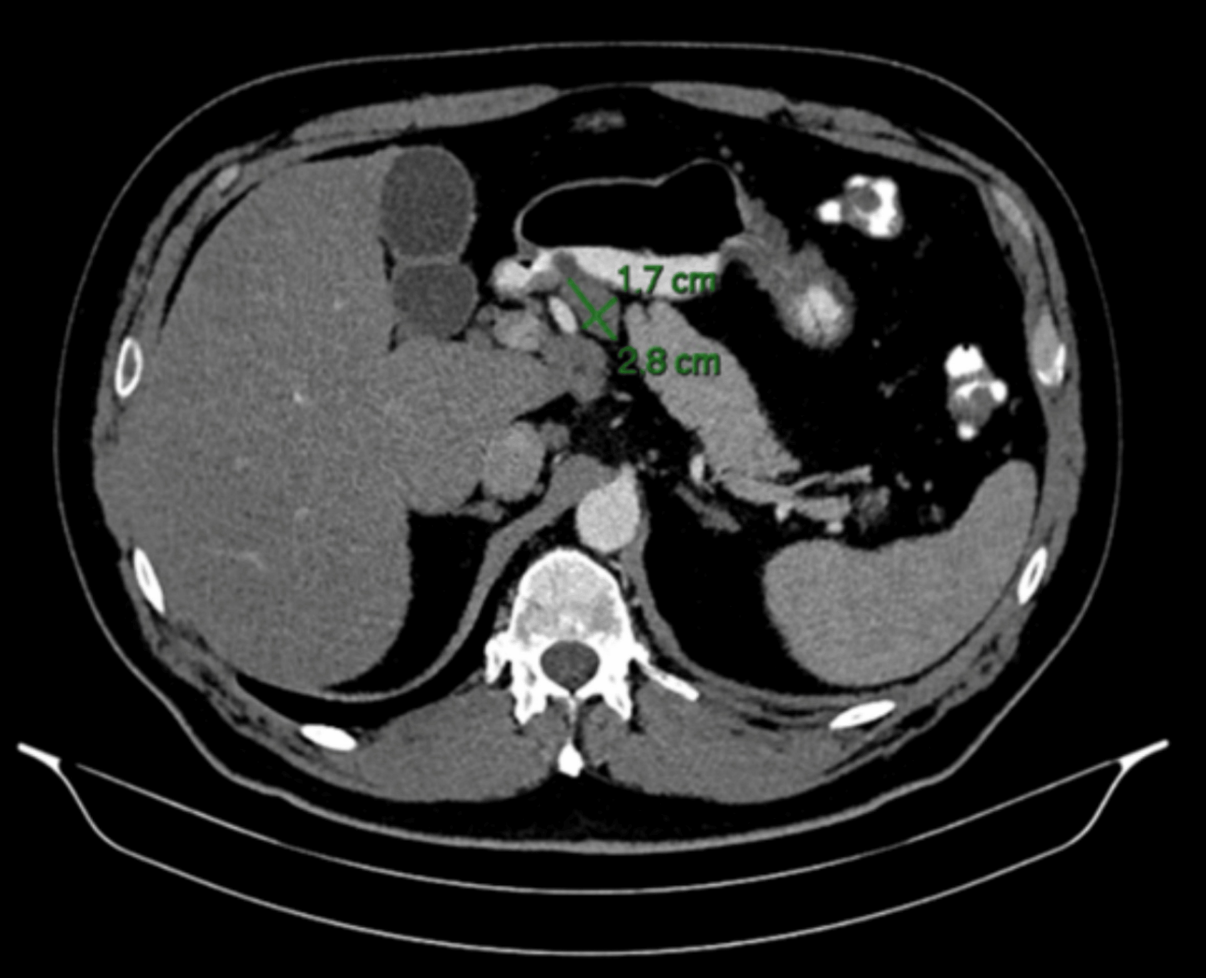
CT abdomen (axial view), showing second prominent lymph node adjacent to the gastric antrum (2.8 × 1.7 cm)

An abdominal CT scan performed at that time demonstrated focal nephronia (Figure 1) involving the right kidney, consistent with acute right-sided pyelonephritis, as well as prominent upper abdominal lymph nodes, including a 2.6 × 2.0 cm node adjacent to the pancreatic head and a 2.8 × 1.7 cm node near the gastric antrum (Figures 2, 3). The pancreas, spleen, CBD and surrounding abdominal organs appeared normal, with no abnormal masses, collections or structural abnormalities identified. He was treated with antibiotics, resulting in subsequent clinical improvement. In view of his metabolic history and deranged LFTs, a FibroScan was performed, which showed an elevated controlled attenuation parameter score consistent with MASLD.

**Figure 4:**
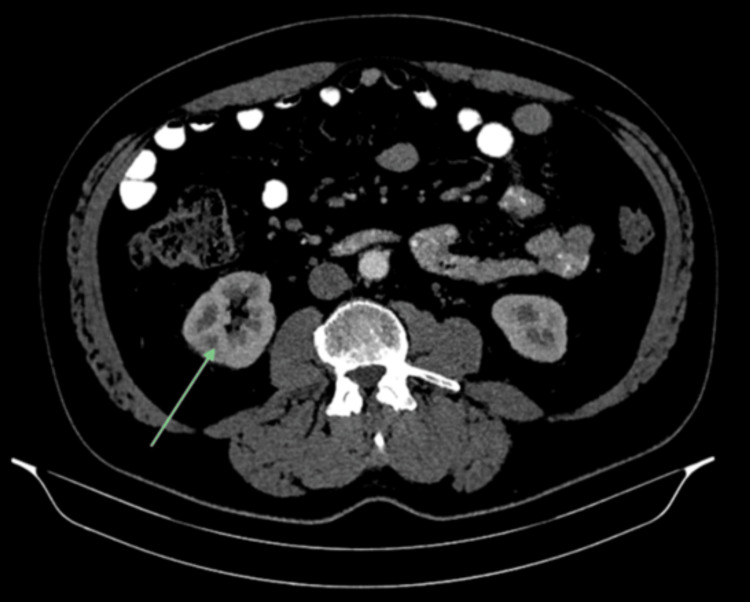
Follow-up CT abdomen, showing resolution of the right-sided focal nephronia with preserved renal parenchymal enhancement

**Figure 5:**
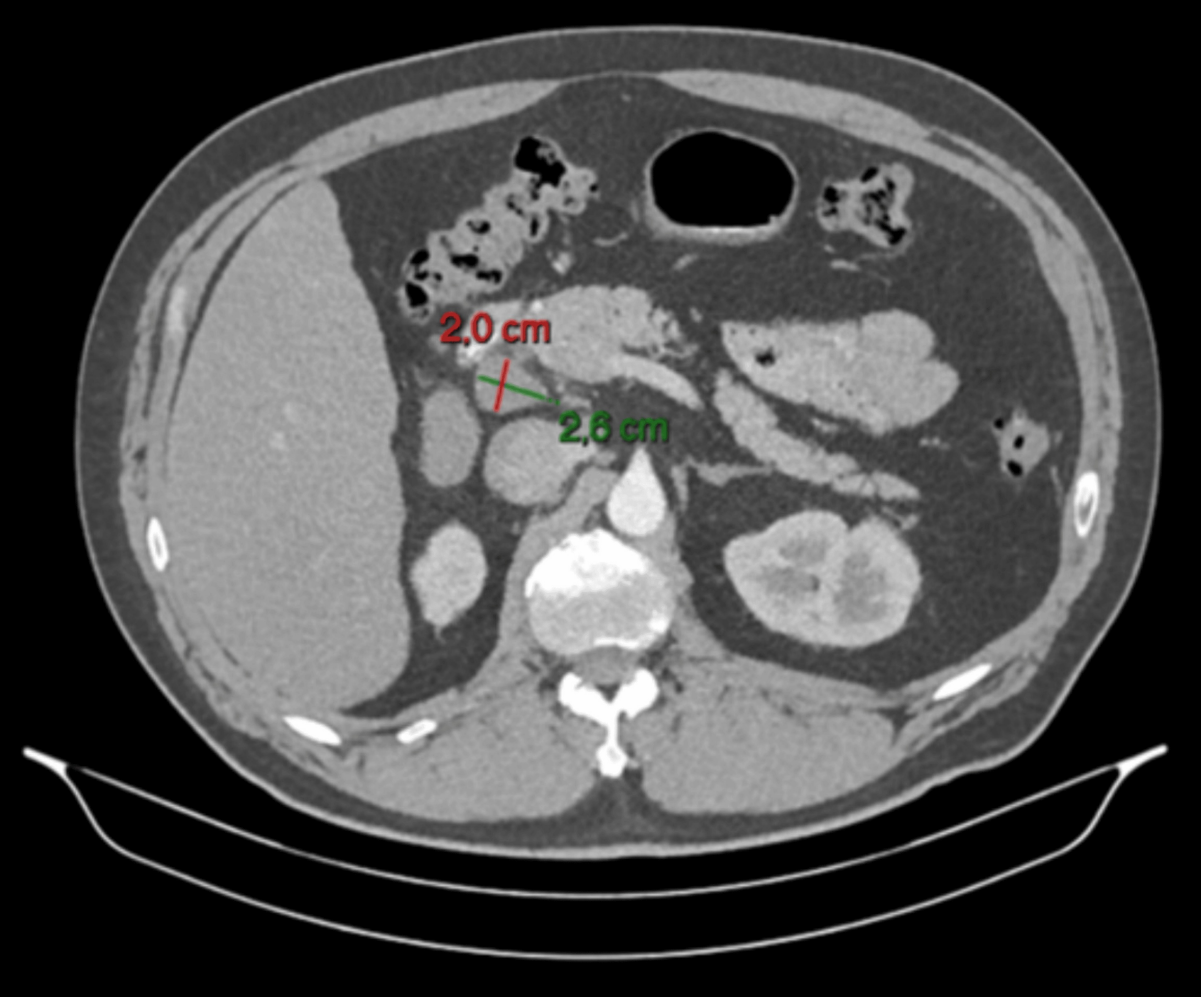
Follow-up CT abdomen (axial view), showing persistent lymph node adjacent to the pancreatic head (2.6 × 2.0 cm), unchanged in size

**Figure 6:**
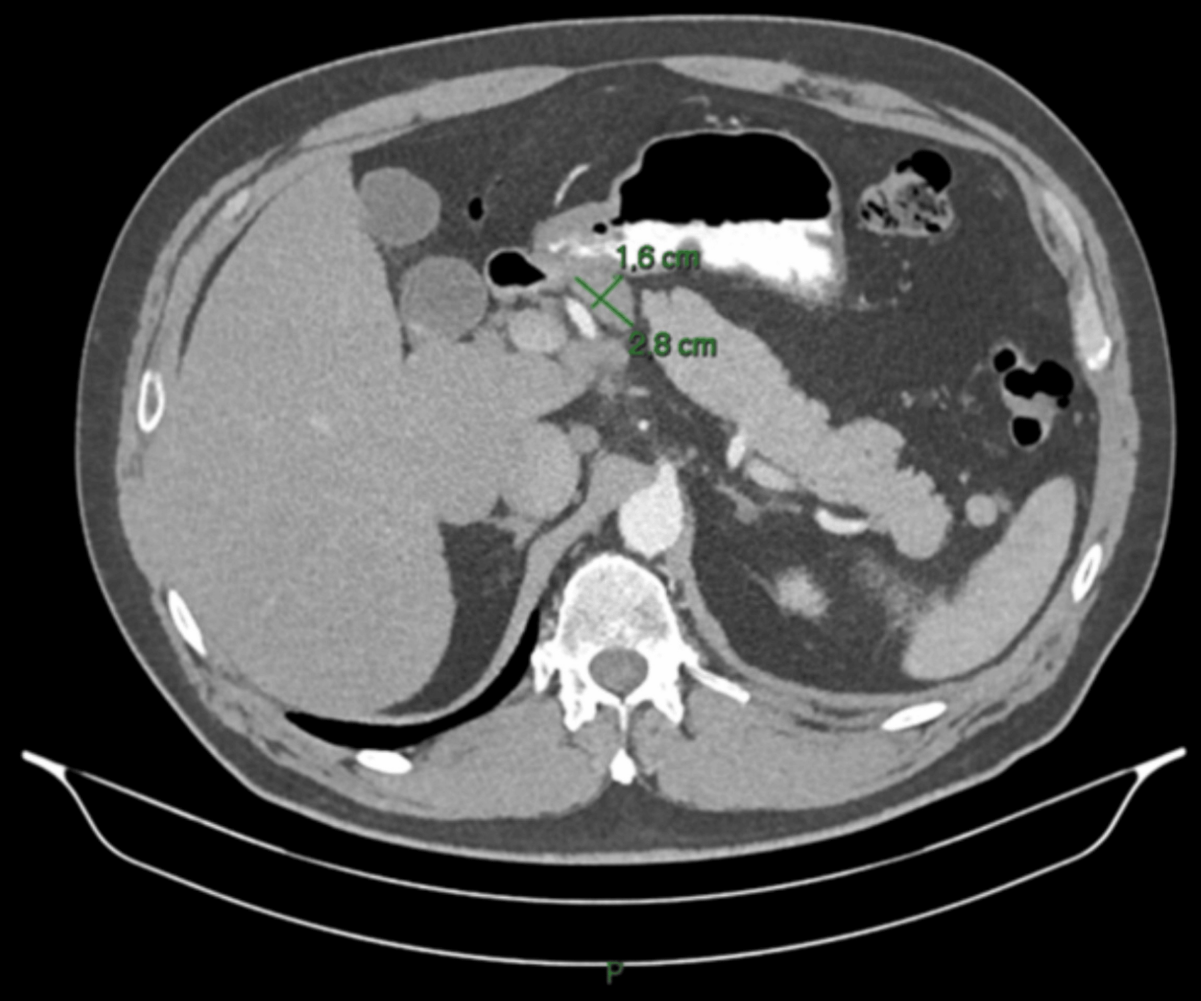
Follow-up CT abdomen (axial view), showing persistent lymph node near the gastric antrum (2.8 × 1.6 cm)

A repeat CT scan, performed three months later, showed resolution of the lobar nephronia (Figure 4); however, persistent lymphadenopathy was noted, including a lymph node adjacent to the head of the pancreas measuring 2.6 × 2.0 cm and another adjacent to the gastric antrum measuring 2.8 × 1.6 cm (Figures 5, 6), with no significant change in size compared to prior imaging. Given the stability of these nodes despite infection resolution, the possibility of a non-infective aetiology, such as reactive or metabolic inflammation, was considered. In view of these findings, he was referred for EUS.

**Figure 7:**
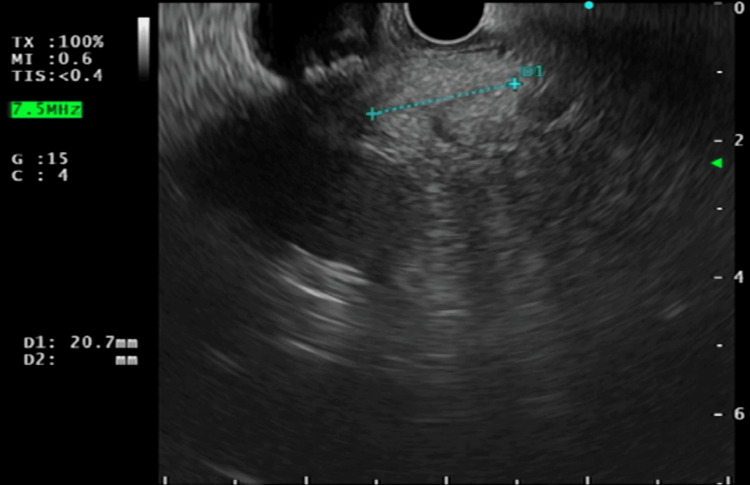
Endoscopic ultrasound image showing a peri-pancreatic lymph node (20.7 mm) in cross section

**Figure 8:**
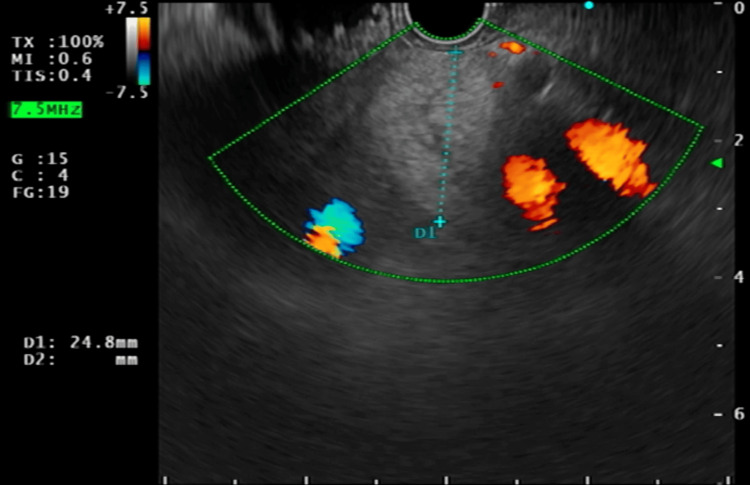
Endoscopic ultrasound image demonstrating a peri-pancreatic lymph node (24.8 mm) with colour Doppler flow

EUS demonstrated a homogeneous, echogenic oval peri-pancreatic lymph node adjacent to the head of the pancreas, measuring 24.8 mm (Figure 7). Using colour Doppler imaging, the lymph node was clearly delineated from the surrounding vascular structures (Figure 8).

**Figure 9:**
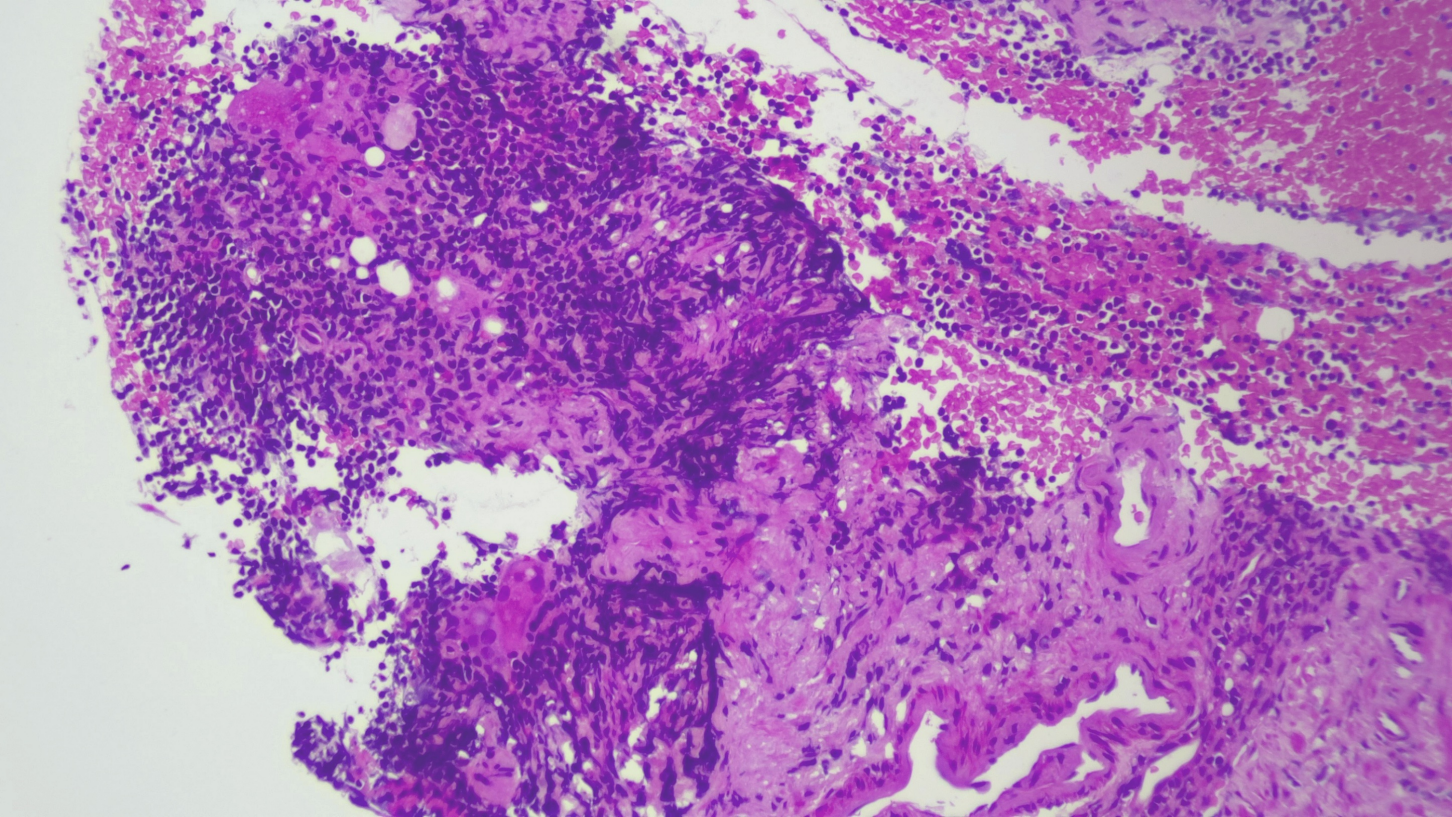
Histopathological examination showcasing features of lipogranulomatous inflammation

**Figure 10:**
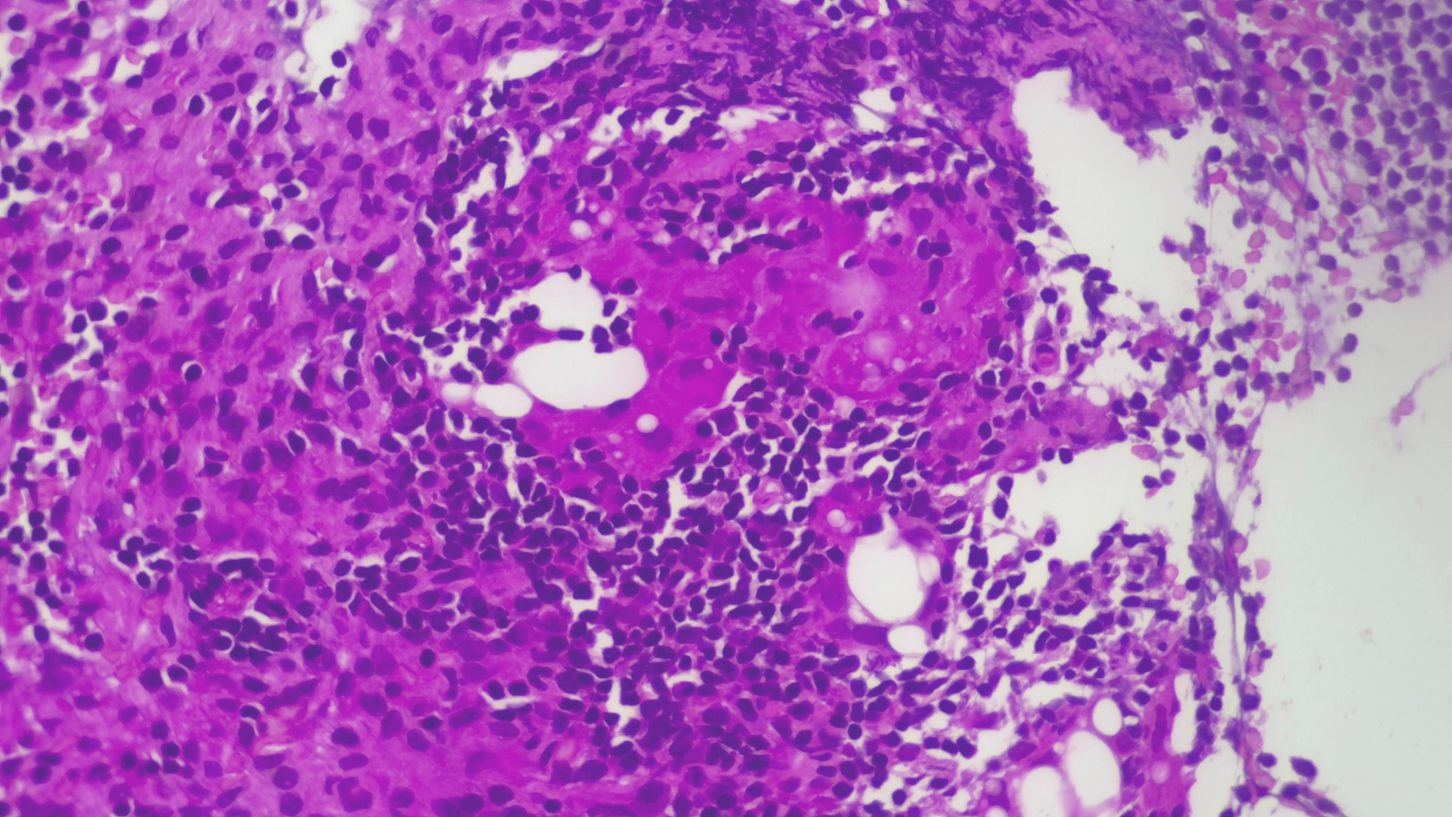
Histopathological examination showing lymph node parenchyma containing adipocytes, some of which are engulfed by multinucleated giant cells

Tissue sampling was performed using a 22-G fine-needle biopsy needle, without immediate complications. Histological examination revealed features of lipogranulomatous inflammation, with no evidence of malignancy or granulomatous infection. Microscopic evaluation showed lymph node parenchyma containing adipocytes, some of which were engulfed by multinucleated giant cells, consistent with lipogranulomatous inflammation (Figures 9, 10). Periodic acid-Schiff and Ziehl-Neelsen stains were negative for fungal organisms and acid-fast bacilli, respectively.

Following the procedure, the patient remained clinically stable and was discharged on the same day. He was subsequently counselled on the benign nature of the findings and the potential association with his underlying metabolic risk factors. Supportive management for MASLD, including dietary modification, weight reduction and optimisation of cardiovascular risk factors, was recommended. Interval follow-up with repeat cross-sectional imaging at six months was advised to ensure stability or resolution of the lymphadenopathy.

## Discussion

Lipogranulomatous inflammation is a rare histological pattern characterised by the accumulation of lipid-laden macrophages (lipophages), multinucleated giant cells and chronic inflammatory infiltrates, often in response to fat necrosis or lipid deposition [1,3].

The defining histological features of lipogranulomatous inflammation include an accumulation of foamy histiocytes (lipid-laden macrophages), multinucleated giant cells, lymphocytic infiltration and fat necrosis. In some cases, such as those described by Jimenez-Heffernan et al., the presence of multinucleated giant cells with cytoplasmic vacuoles and active phagocytosis of lipid droplets has been documented [3]. Such features may easily be misinterpreted as malignant or infectious granulomatous disease if not considered in conjunction with clinical findings. Additionally, fat necrosis and haemorrhage can occur as secondary changes following biopsy or trauma, especially in lymph nodes sampled near fatty structures such as the breast or mesentery [3].

The differential diagnosis of lipogranulomatous inflammation is broad and must include both neoplastic and non-neoplastic causes. Sarcoid-like granulomas have been described in lymph nodes of patients with Hodgkin’s disease and may mimic lipogranulomatous patterns [4]. Moreover, lipid lymphadenopathy has previously been associated with reactions to oily radiographic contrast media used in lymphangiography. This was an important historical consideration, although such agents are now rarely used [4].

Endogenous lipid release, often due to trauma, necrosis or metabolic derangement, is also implicated. George and Issam, for instance, highlighted multiple endogenous triggers, including fat embolism, necrotic fat and lipid-rich tumours [1]. In one case, gluteal intermuscular injections of lipid-based drugs resulted in lipogranulomatous lymphadenitis, emphasising the role of exogenous lipids as aetiological agents [1].

In addition to the above, infectious aetiologies such as Whipple's disease must also be considered. Although rare, this condition can involve abdominal lymph nodes and demonstrate histiocytic infiltrates that may resemble lipogranulomatous inflammation [5,6]. The sinuses of involved nodes often show expansion with lipid-laden macrophages, and other atypical mimics include *Mycobacterium avium-intracellulare*, silicone lymphadenopathy and lysosomal storage diseases [6].

Beyond human cases, veterinary literature has documented similar patterns. For example, Van Kruiningen et al. and Watson et al. described lipogranulomatous lymphangitis in dogs with protein-losing enteropathy and obstructed mesenteric lymphatics [7,8]. While not directly translatable to human pathology, these models support the role of lymphatic stasis and lipid accumulation in the pathogenesis.

In endoscopy, lymph node biopsies are most often pursued to rule out malignancy and tuberculosis, among other causes. The identification of a benign but rare pattern, such as lipogranulomatous inflammation, may therefore be confusing or dismissed as non-specific. Awareness of this entity, particularly in patients with MASLD and imaging evidence of periportal lymphadenopathy, may help guide appropriate management and prevent unnecessary escalation.

Portal lymphadenopathy is a known radiological feature associated with non-alcoholic steatohepatitis, the more aggressive phenotype of MASLD [9]. In a large imaging study by Daher et al., the presence of portal lymphadenopathy correlated significantly with histological markers of disease severity, including hepatocyte ballooning and advanced fibrosis. While that study did not evaluate the histopathological features of the lymph nodes, our case highlights that lipogranulomatous inflammation may be a possible histological finding in patients with MASLD.

## Conclusions

This case highlights lipogranulomatous inflammation as an unusual but benign cause of peri-pancreatic lymphadenopathy detected on EUS. Although uncommon, this entity can closely mimic malignant or granulomatous disease both radiologically and histologically, underscoring the need for careful clinicopathological correlation. Awareness of lipogranulomatous inflammation can help clinicians avoid unnecessary interventions, guide appropriate patient counselling and broaden the differential diagnosis of hyperechoic lymph nodes encountered during EUS. However, the conclusions drawn from this single case should be interpreted with caution, and further studies are warranted to validate these observations before broader generalisation. However, it remains important to recognise the limitations of a single-case observation and the need for further research to substantiate these findings.
